# Engagement of People With Lived Experience in Spinal Cord Injury to Address Innovation Priorities

**DOI:** 10.1111/hex.70369

**Published:** 2025-08-22

**Authors:** Julia T. Ross, Vanessa K. Noonan, John Chernesky, James Hektner, Richard Peter, Spring Hawes, Andrew Forshner, Shweta Shekhar, Tathagata Ray, Arushi Raina, James J. Laskin

**Affiliations:** ^1^ Praxis Spinal Cord Institute 6400‐818W 10th Ave Vancouver British Columbia Canada

**Keywords:** commercialization, engagement, focus groups, innovation, lived experience, spinal cord injury

## Abstract

**Introduction:**

The involvement of people with lived experience (PLEX) of spinal cord injury (SCI) is not common during medical innovation design, research and commercialization processes. To address the SCI community's needs and priorities, companies developing SCI‐related innovations must include individuals living with SCI in all steps of the process. This study explores the importance of the involvement of PLEX groups in identifying key themes and priorities for SCI‐related devices and therapeutics.

**Methods:**

Focus groups were held virtually as part of the Praxis Innovation Program. Members of the Praxis PLEX team facilitated 90‐min discussions with PLEX participants. A discussion guide was used which included the following topics: existing challenges & solutions, initial product impressions, likelihood of use, acceptance by SCI community, reimbursement/payment challenges & solutions, and potential improvements. Researchers observed the focus groups and took contemporaneous notes. Following each focus group, a deidentified summary was synthesized using researchers' notes, facilitator's key messages, and an audio transcript. The summaries from all focus groups were analyzed and themes common across the focus groups were identified.

**Results:**

Twenty‐seven focus groups were conducted between 2020 and 2023, with an average of seven participants per session. Individuals from Canada, the United States, New Zealand, and Australia participated. Participants ranged in age from 21 to 73 years. 58% were male and 42% were female. Fourteen percent of participants had lived with SCI for less than 5 years, 17% for 6–10 years, 12% for 11–15 years, and 57% for greater than 16 years. Among participants, 59% were tetraplegic, while 41% were paraplegic. Six global themes emerged that reflected PLEX's considerations for new technologies; purchase and use decisions were identified in 96% of the focus groups, purpose in 93%, design in 89%, communication in 85%, time factors in 81%, and adaptation considerations in 81%.

**Conclusion:**

This study describes the results of using a systematic approach and framework for SCI PLEX focus groups during the research, design and commercialization process. The themes identified: purchase and use decisions, purpose, design, communication, time factors, and adaptation considerations, are important topics regarding SCI innovations. Future studies should focus on measuring the impact of patient/PLEX engagement by comparing the success of devices that successfully reach consumer market after collaboration with PLEX versus products that are developed independently.

**PLEX and or Public Contribution:**

Members of the Praxis PLEX team provided significant feedback and input on developing the focus group discussion guides, facilitating the focus groups, interpreting the data, and producing the manuscript. They were all included as co‐authors.

## 1 Introduction

Spinal cord injury (SCI) is a life‐altering neurological condition that has a significant socioeconomic impact on patients, their families and society [1]. Impairment from the injury is determined by the severity and location of the lesion along the spinal cord [2]. The global incidence of SCI is estimated to be between 40 and 80 cases per million population [3], while the prevalence is estimated to be 20.6 million cases worldwide [4]. In addition to injury to the spinal cord, many of the body's systems are impacted due to damage to the voluntary and autonomic nervous systems. With no cure for SCI yet available, living with a SCI is a chronic health condition. While life expectancy after SCI is lower than in persons without SCI, many individuals live decades with their injury and medical devices are required to manage their health and provide autonomy so they can meaningfully participate in society [5].

The International Classification of Functioning, Disability and Health (ICF) is a biopsychological framework that considers how an individual's medical diagnosis, personal factors, and the environment impact the individual's ability to function [6]. Under the ICF framework, medical devices are categorized as environmental factors that can modify the disability experience for individuals living with SCI. These devices can enhance autonomy, improve health and quality of life, and decrease the cost of care following SCI. Previous studies asking people with lived experience of SCI about their priorities for health research and innovation have identified that upper extremity function is the highest priority for individuals with tetraplegia (loss of motor and sensation in upper and lower extremities) [7], while bowel, bladder and sexual functioning are rated highly for people living with paraplegia (loss of motor and sensation in the lower extremities) [8].

Along with identifying priority areas for research, patient engagement is critical in ensuring that SCI medical innovations address the priorities, wants and needs of the SCI community. In this paper, we adapted the definition of patient engagement from Harrington et al.'s 2020 paper which proposes the following definition for patient engagement in research: “The active, meaningful, and collaborative interaction between patients and researchers across all stages of the research process, where research decision making is guided by patients' contributions as partners, recognizing their specific experiences, values, and expertise” [9]. We used the term innovation rather than research as the process in which engagement occurs, and extended the term patient to include PLEX, which reflects a broad range of users.

PLEX engagement has been part of some SCI preclinical [10] and rehabilitation research [11], and designing clinical care [12]. The involvement of lived experience corresponds with the “living lab” approach which centers on innovation in collaboration with end‐users [13], and has been shown to improve healthcare innovation implementation outcomes [14]. While living labs are increasing in popularity, their use in SCI innovation development has not been commonly described in the scientific literature [15].

In alignment with the living lab philosophy, Praxis Spinal Cord Institute launched the Innovation Program to facilitate the translation of innovations that consider PLEX priorities. The Program consists of SCI Incubate and SCI Accelerate, which support early and late‐stage innovations respectively (https://praxisinstitute.org/innovation). The Praxis Innovation Program utilizes an Integrated Knowledge Translation (IKT) [16] approach, combining experts from Praxis' Research, Clinical, and PLEX teams. Key features of the Innovation Program are the PLEX focus groups, facilitated by members of the Praxis PLEX team, with participants from the global SCI community. These focus groups provide feedback to help innovators refine their products based on the needs and priorities of PLEX. This paper aims to identify the themes expressed by the PLEX focus group participants on emerging SCI innovations and share the learnings from PLEX involvement in SCI innovation.

## 2 Methods

### 2.1 Focus Group Study Design

The Praxis Innovation team collaborated with the Praxis PLEX, Research, and Clinical teams to conduct the focus groups. The focus groups were conducted in the second month of the Innovation Programs to provide adequate time for the companies to refine their products based on the feedback from the focus groups before the conclusion of the program. The focus groups were held virtually using a video conferencing platform to optimize participant availability and accessibility. For each focus group, members of the Praxis PLEX team led a 90‐min group discussion in English. Members of the companies were not present in the focus group to encourage the participants to speak freely and provide honest feedback free of influence from the company. After the focus group session, participants received an honorarium in recognition for their time and expertise. The honorarium amount (approximately $75 Canadian dollars) was chosen as an incentive to enhance recruitment, without undue coercion [17].

A generic focus group discussion guide template was developed, as shown in Table 1. Based on this template, a company‐specific discussion guide was developed in collaboration with the company, the clinical researcher, the PLEX team lead working with the company, and the Innovation team. The discussion guide included open‐ended questions, allowing the facilitators to introduce prompts and follow‐up questions as needed. The goal of the discussion guide template was to ensure that the focus group addressed questions that the companies wanted covered for its product development.

**Table 1:** Generic discussion guide.

Discussion topics	Questions	Probes
Existing challenges & solutions	What are some current challenges to… [insert topic area]	How are they being solved (how could they be solved)?
Initial product impressions	What are your initial impressions of this product? Why is this?	Thinking about challenges/solutions – where/how does this product fit in?
		Does it address one of the challenges? Is it a solution?
Likelihood of use	How likely do you think you would be to use it (once it becomes available)?	Very/Somewhat/Not Much/Not at All
		More likely to use because… Less likely to use because…
		[specific probes based on product]
Acceptance by SCI community	How likely do you think others with spinal cord injuries would be to use it? Why is this?	What about other health conditions?
Reimbursement/payment challenges & solutions	Is this product similar to another which you or your [medical services] funder already pays for or reimburses?	If yes, which one(s)? How much do they cost/are they reimbursed for?
	How much do you think you or your [medical services] funder would be willing to pay for this product? Why is this?	What could be done to make it easier for you or your [medical services] funder to pay/reimburse for this product?
Potential improvements	What changes or additions to this product could be considered?	How high a priority would you give some of those we have already talked about, for example…
Other	Is there anything else you would like to add?	

### 2.2 Participant Recruitment and Selection

Purposive recruitment, a nonrandom qualitative research sampling method used to identify and select participants with “information‐rich” experiences in the interest area [18], was done via criterion sampling. Recruitment emails were sent to individuals on Praxis' PLEX contact lists, and informational posts were placed on several social media platforms. All potential participants were directed to an electronic consent form and application form. Once consent was provided, interested individuals filled out the online application form consisting of their name, birth year, sex at birth, gender, home location, contact information, number of years living with SCI, level of SCI, non‐traumatic or traumatic, and severity of injury. Lastly, the applicant was provided with a short explanation of each company, focus group inclusion‐exclusion criteria, and the times/dates of the 90‐min sessions. Finally, the applicant was asked to rank their focus groups of interest. Potential participants who were not on the Praxis contact list or not known to the PLEX team were then verified using a video call to ensure the participant was appropriate for the focus group.

Potential participants were identified from the pool of applications and invited to participate via email. The goal was to have a minimum of six individuals attending each focus group to ensure heterogeneity. Ultimately, 10 to 12 individuals were invited to each focus group, in case some individuals selected to participate were not able to attend the focus group. A nondisclosure agreement was signed by participants to ensure the confidentiality of the company's innovation. Attached to the invitation email was a brief company information document and short informational video, which each company developed in collaboration with Praxis. The participants were asked to review the materials before the focus group session. Twenty‐four hours before the session, a reminder email was sent to each participant. A Consolidated Criteria for Reporting Qualitative Research (COREQ) checklist is included in Appendix S1 for further study details.

### 2.3 Focus Group Sessions

The focus group sessions were initiated on Zoom. Before being allowed into the session, participants were validated in the Zoom waiting room, and each participant was asked to ensure that only their first name appeared in the window associated with their image. Once all the participants arrived, the facilitator welcomed them, reviewed the focus group etiquette, reinforced the importance of participation confidentiality, and proceeded with introductions. During the following 90 min, the facilitator worked through the company‐specific discussion guide. After the session, the facilitator thanked the participants for their time, comments and opinions and reminded them about participant confidentiality.

### 2.4 Data Collection

Three to four researchers collected data during the focus groups, two of whom, the principal investigator and program director, were present for every focus group. The researchers took contemporaneous notes while they observed with their microphones muted and videos off. During the first year of the Innovation Program, a professional transcriber produced deidentified transcripts. In subsequent years, the meetings were recorded, and an automated transcription technology, Otter AI (Mountain View, CA), used the recordings to create transcripts. Once the transcripts were complete, the recordings were destroyed.

### 2.5 Ethics

The recruitment protocol, focus group methodology, and informed consent used for the SCI Accelerate and SCI Incubate Programs were approved by the Veritas Independent Review Board (Kirkland, Quebec, Canada): 2023‐2480‐16041‐3.

### 2.6 Data Analysis

Following each focus group, a detailed and deidentified executive summary was written by an Innovation team member. The summary synthesized the researchers' notes, the facilitator's key points and the transcript. The summary included the participants' basic demographic information and identified significant themes and direct quotes from the discussion that highlighted the themes. The facilitator and another Innovation team member reviewed the focus group summary to ensure accuracy and anonymity. Finally, the summary was reviewed by the researchers who observed the focus group.

After the focus groups were completed, a researcher who did not participate in the Praxis Innovation Programs performed a thematic analysis on the focus group summaries. Key words and topics were coded manually and data saturation of the codes was used to identify the themes. The instance of each theme was summed. To ensure data saturation, a defined threshold of 80% was used and themes present in over 80% of the summaries became global themes. Once the global themes were identified, a researcher who was involved in the focus groups reviewed them for accuracy.

## 3 Results

### 3.1 Participants

There were 27 focus groups (see Table 2), of which 12 were in the SCI Incubate Program, and 15 were in the SCI Accelerate Program. The focus groups had an average of 7 participants per session, a minimum of 4, and a maximum of 12, resulting in a total of 198 participants. Of the 198, 4 participants had a non‐traumatic SCI, and the remainder had a traumatic SCI. Participants ranged in age from 21 to 73 years, and 58 percent were male and 42 percent female. Fourteen percent of participants had lived with SCI for less than 5 years, 17 percent had between 6 and 10 years, 12 percent had between 11 and 15, and 57 percent had greater than 16 years of experience. Among the participants, 59 percent were tetraplegic, and 41 percent were paraplegic. Individuals from Canada, the United States of America, New Zealand, and Australia participated. Individuals were allowed to participate in multiple focus groups, yet over 75% of participants were unique. To preserve the anonymity of the contributors, participant identifiers were not included when reporting quotes from the focus group discussions.

**Table 2:** Focus group details.

Product—recovery target area^a^	Number of	Average number
	focus groups	of participants
Bowel/bladder assessment/management	5	7
Lower extremity function/rehabilitation	1	7
Motor recovery (ex. electrical stimulation)	2	8
Neuropathic pain	1	7
Pressure injury/wound management	6	7
Sexual function	1	4
Spasticity management	1	6
Spinal cord repair (ex. pharmaceutical, surgical)	2	9
Temperature regulation	1	12
Upper extremity function/rehabilitation	5	7
Wheeled mobility	2	8

^a^ Targeted areas of recovery for the devices and therapeutics involved in the Praxis Innovation Programs and discussed in the focus groups. Target areas were chosen by the companies and not PLEX.

**Figure 1:**
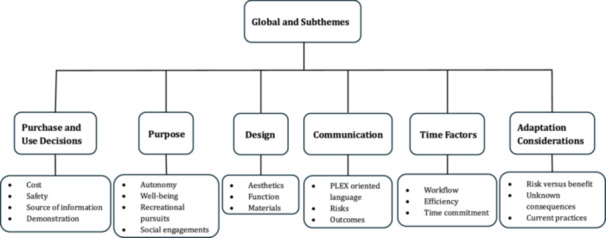
Global themes and sub‐themes.

The innovations discussed in the focus groups ranged from assessment, rehabilitation, and management devices to pharmaceutical and surgical devices. While the structure of each focus group was similar, the specific questions asked varied across each focus group depending on the innovation. Overall, six global themes emerged that addressed the SCI recovery priorities. These themes were: purchase and use decisions, which were identified in 96% of the focus groups, purpose in 93%, design in 89%, communication in 85%, time factors in 81%, and adaptation considerations in 81%. Along with the global themes were several sub‐themes, as seen in Figure 1 and Table 3.

**Table 3:** Global themes and sub‐themes.

Global theme	Sub‐themes	Explanation	Quote
Purchase and use decisions	Cost	The out‐of‐pocket cost and sources of coverage	“The current cost is prohibitive at the end‐user level.”
	Safety	Evidence of safety from the manufacturer and the community	“Safe, useful, effective. No one is going to spend a bunch of money … if they don't know that it would matter.”
	Source of information	Trusted sources of information influence purchase and use decisions	“[I] go by what my peers, doctors, and experts say or recommend.”
	Demonstration	Access must be available before the purchase decision to ensure functionality	“I would need to [try it] before purchasing or [hear] from someone who is similar to me in terms of level of injury.”
Purpose	Autonomy	Individuals with SCI view autonomy as having greater self‐regulation and independence in their daily lives	“If I can add one thing [to increase my autonomy]… It's a big deal.”
	Well‐being	Aspects of mental, physical, and sexual health	“Mental health aspect should be considered in treatment.”
	Recreational pursuits	Activities and hobbies	“I see it being useful for recreational therapy. It will be useful for crafting.”
	Social engagements	Community and familial engagement	“I hated that everyone had to crouch down… everybody's leaning down to try and talk to you, and it would be nice to be able to be at the same height as everybody and look them in the eyes.”
Design	Aesthetics	Look, color, and shape	“The point is feeling good about yourself.”
	Function	Weight, size, and operational aspects	“It all comes down to function and if I can use it.”
	Materials	Skin sensitivity and packaging	“Worried about pressure sores with sweating and sensitive skin.” “The garbage and the landfill that would outlive me. That's the one thing I have an issue with.”
Communication	PLEX‐oriented language	Appropriate information intended for the end user	“I like the one‐pager… clear, simple to read and follow. Impactful with the visuals and hit all the key points.”
	Risks	Transparent communication about side effects, potential risks, and clinical trial results	“Everyone is interested in the side effects and cross effects.”
	Outcomes	Evidence of usefulness, benefits, and effectiveness from the manufacturer and community differentiating the innovation from current devices	“…willing to try once I hear someone I know has tried it with long‐term experience.”
Time factors	Workflow	The process or steps taken to complete a given task or activity	“Would be a game changer for me… it would simplify almost everything I try to accomplish.”
	Efficiency	Individuals with SCI face a scarcity of time due to the increased time allocated to managing their injury and secondary complications	“It was life‐changing for me. It took 10 min off every time I had to go pee… I didn't think that was possible.”
	Time commitment	Location and frequency of required external care	“In most cases, I would prefer not going to the doctor…It takes extra time to go to the doctor.”
Adaptation considerations	Risk versus benefit	Analysis of the potential risks and the anticipated benefits	“It's just the pros and cons, right? If the [procedure] doesn't work, what could you be losing?”
	Unknown consequences	Possible unanticipated results, side effects or complications related to new device adoption	“It's scary to change up your routine.”
	Current practice	While the current device may not be the best, it works for the individual	“To convince people to come away from what they've been using for 20 years will be another potential hurdle.”

*Note:* Prevalent themes identified from the focus groups: Purchase and Use Decisions, Purpose, Design, Communication, Time Factors, and Adaptation Considerations and their sub‐themes, explanations, and quotes.

### 3.2 Purchase and Use Decisions

The first central theme identified was purchase and use decisions. Participants expressed that “cost is critical” and had many questions about price points and reimbursement.

The high out‐of‐pocket cost was identified as a barrier to purchase.

Those on disability benefits cannot afford basic amenities like Wi‐Fi and gym [fees] due to inflation and low amount [of] benefits.

They noted that while upfront costs often seem large, specific therapies could reduce healthcare costs over a lifetime if they reduced the required care.

I'm 100% dependent on caregivers for everything. So, lowering the hours of the day I need care, I mean over my life expectancy… I think right now it's two to three million estimated, but with inflation, that's going to go up to five or six million.

Participants said they would be more likely to consider adoption if their insurance covered the innovation. However, some individuals felt that if the device significantly improved quality of life, the cost would be justified regardless of the price.

Well, let's be honest here; it's our lives we're dealing with.

Additionally, participants unanimously felt that demonstrated safety was a priority for adoption. In particular, if there was a perceived safety concern or the device or procedure was invasive, there was hesitancy about being an early adopter.

Participants also highlighted the importance of the source of information regarding who was promoting the innovation.

I only talk to my local equipment supplier who really knows their stuff and is also someone with lived experience.

While the PLEX valued the data provided by the manufacturer and healthcare professionals, they were potentially more interested in what their peers shared via social media.

Whenever you find something that works, you can count on people telling each other.

There was a consensus that PLEX would like the opportunity to demo the product before making their adoption decision. A trial period allows potential buyers to touch and feel the product, thus determining how the device would fit into their daily workflow while confirming perceived efficacy and safety. Confirming that the product had the advertised function would promote adoption.

So, to just purchase this at today's stage, I don't think I'm quite there yet, but [during] a demo period, I could maybe, really appreciate what the product is and what it has to offer.

Overall, the combination of cost, safety, sources of information, and opportunities for demonstrations were determined to influence whether an individual would purchase and use a device.

### 3.3 Purpose

The purpose of the device in improving autonomy for individuals in the SCI community cannot be overstated as they described the complexity of daily living with SCI. Autonomy in occupational tasks was mentioned in multiple contexts, including personal hygiene, the ability to don and doff a device, the reduction of required caregiver time, and regaining function.

Being able to do these little things is priceless, empowering.

PLEX conveyed a desire to preserve existing independence through therapies and devices.

… more ability to do things on your own without having to ask or rely on anyone [would] be good. Definitely beneficial.

…a little bit of restoration of function can be very, very valuable from a like reducing care side of things, but also the psychological impact.

The ability to make independent decisions dramatically affects well‐being and mental health.

[I] would love to eat and feed on my own at my own pace. Having people help with feeding takes away from the experience. What is on each spoon/fork ‐ how much, what order. Making these decisions on your own helps you forget about the other daily challenges.

PLEX value innovations that improves their ability to meaningfully participate in activities, recreational pursuits, and social engagements.

I tell people…I'm going to be paralyzed, and I may as well be happy when I'm paralyzed.

Autonomy regarding sexual health was another factor contributing to PLEX's well‐being. A group of male PLEX emphasized the importance of controlling their sexual function to improve both sexual and romantic relationships for themselves and their partners. PLEX felt that constantly having to plan when initiating intercourse resulted in a loss of spontaneity in sexual relations and was a significant relationship challenge.

The SCI community would like spontaneity [in their sex lives].

### 3.4 Design

PLEX preferred discreet devices, and they found aesthetically pleasing devices had psychological benefits.

I got frustrated with having to just wear pants or long skirts or things to cover the leg bag, being that the fashion of the day [is] tights and skinny jeans. I'm like, dammit, I want to figure out how to do that even with my leg bag.

Functionally, PLEX wanted portable, easy‐to‐use devices.

How long does the battery last? … Do I have to pack a charger with me everywhere I go?

PLEX had two significant opinions about materials that would be on their bodies. First, they were worried about skin sensitivity.

My skin [is] super ultra‐sensitive to [dressing] tape.

Second, PLEX had observations about packaging. PLEX wanted to remind companies to accommodate users with limited hand function when designing devices to guarantee ease of use and minimize the need for constant intervention.

Is it difficult to open? A lot of us have trouble with our hands.

Additionally, PLEX wanted packaging designed to minimize waste.

### 3.5 Communication

The focus groups revealed knowledge gaps between companies and PLEX, stemming from inadequate communication, which could lead to confusion or misunderstandings. PLEX emphasized the need for clear, honest, and respectful communication from companies.

PLEX felt that companies could improve by providing PLEX‐oriented instructions or information in addition to the traditional information written for clinicians. Some PLEX found long instructions full of medical jargon overwhelming, stating that consolidated, straightforward information would be more beneficial.

I don't need that many attachments… and also, I might be a little afraid of, like, how can I learn 70 attachments?

PLEX also expressed that during the acute phase of SCI, newly injured patients can be heavily reliant on their families or caregivers to make decisions as PLEX “emotions were so crazy” and medications “affect[ed] you cognitively.” The focus group stressed the importance of clear communication between companies and clinicians to inform decision‐makers who may not have a previous understanding of SCI.

… there's a million different little things that we all know now that we wouldn't have a clue about when we're first injured.

Communication about risk was a significant priority for PLEX who wanted transparent and responsible messaging regarding clinical trial results, side effects, and long‐term effects.

PLEX stated that “having trials increases confidence” but also expressed hesitancy to participate in trials. Participants cited examples where they lacked sufficient information for acute clinical trials, impacting the informed consent process.

Depends [on] who is running the trial, the private company won't be as likely, if it's ICORD [International Collaboration on Repair Discoveries], maybe. If it's a venture capital firm, no.

Most of the PLEX questions were about side effects, long‐term effects, risks, and benefits.

I don't know that I would be the first to try it. But I know after […] got it and tried it… word would get out because nothing is sacred in our community… you hear success stories around the community.

Additionally, PLEX felt that communication from personal connections would enhance their sense of safety and trust in the information provided.

I wouldn't be here today if it wasn't through Praxis and the people who invited me here because I know them; they have credibility.

Clinical trial results and user experiences were data that PLEX trusted.

All of the literature has to be made available… Some of this stuff is pretty complex. You need to be able to read the statistics and have people explain to you how the statistics are working.

Additionally, PLEX noted that many of the devices would be used in a clinical setting; therefore, the results and benefits would also have to be advertised to clinicians.

They [clinicians] want to be able to see data that [the device] is going to benefit their patients above and beyond the conventional rehab that they already provide.

Companies must also properly communicate how their product differs from the pre‐existing devices. PLEX were less interested in devices similar to their current devices regarding outcomes and benefits.

Occasionally, it was noted that dissemination was necessary for companies to address misconceptions about their technologies. PLEX reacted negatively when they needed help understanding the intended outcome feeling that the device was not effective enough.

### 3.6 Time Factors

Individuals with SCI struggle with limited time as daily tasks become more complicated and time‐consuming. As one PLEX explained, “There's enough headaches going through this SCI thing every single day,” and another mentioned it took “an hour to shower.” PLEX wanted innovations that reduced steps, simplified their workflow, and improved efficiency to maximize their time in a day.

You want the process to be super quick, super easy, and less messy.

PLEX were also interested in local interventions and therapies that reduced their time commitments.

I travel so much around town for the different treatments and to see different doctors and things we have to do anyway… The more it can be compressed, the better.

PLEX noted that getting a prescription from a doctor could produce a barrier to access and delay treatment.

I'm saying if we have to go to our doctor, it's just another delay [for treatment].

### 3.7 Adaptation Considerations

The risk versus benefit comparison was commonly brought up. The level of injury could impact decisions as some PLEX felt higher‐level injuries would be more inclined to accept greater risk. However, some PLEX noted that lower‐level injuries were more risk‐tolerant.

During different stages of SCI, PLEX also noted that their priorities shifted. In the acute phase, PLEX remembered being willing to risk anything if it offered potential benefits or recovery.

I would say when I was first injured, there's no doubt I would have tried anything at that time.

Meanwhile, in sub‐acute and chronic phases PLEX noted that their priorities shifted from walking to autonomic functions.

Bladder and bowel function wasn't something that I considered [initially].

Unintended consequences, such as interventions causing increased pain or spasms, were possible outcomes that some PLEX were not willing to risk.

PLEX discussed how changing from their well‐known routine was scary. Although some PLEX were quick to buy into new innovations, others were more wary.

It is the users, the users' comments, the long‐term proof that it's helping your same type person, you know, user experience comments would potentially help convince those who don't buy in as quickly.

## 4 Discussion

Praxis Innovation Programs aim to facilitate the translation of innovation from ideation to market so that individuals living with SCI can benefit from those innovations. Between 2020 and 2023, 27 focus groups generated six important themes to consider when developing solutions for individuals living with SCI. The participants included PLEX of different sexes/genders, ages, years of injury, severity of injury, and level of injury. Participants discussed innovations ranging from noninvasive daily care devices to drug development and invasive, potentially ground‐breaking cure technologies. Despite the diversity in innovation type, the themes that emerged from each focus group were consistent.

The most dominant theme was purchase and use decisions, which was present in 96% of the focus groups. The theme was further divided into sub‐themes based on aspects of the device that influenced participant's decisions: cost, safety, source of information and demonstration. While health innovation can potentially augment rehabilitation outcomes [19], it has also been reported that consumer perception of the usefulness of assistive devices is significantly related to device use [20]. In Canada, many basic equipment costs are covered by provincial funding programs or private extended healthcare programs, but there are many out‐of‐pocket costs for equipment deemed non‐essential [21]. Often, equipment costs are paid by the consumer as many countries have insufficient public funding, and as a result, cost and potential reimbursement constitute a significant concern for PLEX [22].

Purpose was the second most prevalent theme, present in 93% of the focus groups, and was divided into four sub‐themes: autonomy, well‐being, recreational pursuits, and social engagement. Autonomy is fundamental in health conditions such as SCI, where a person suddenly loses independence [23]. Innovation can play an important role by enabling individuals to be less reliant on help, allowing them to return to meaningful participation in activities such as work, social events and recreation, which tremendously impact the quality of life [24]. Gómara‐Toldra reported that participation has a stronger association with subjective quality of life than physical impairments and activity limitations for individuals with SCI [25].

The next theme was design, which appeared in 89% of the focus groups and was divided into three sub‐themes: aesthetics, function, and materials. Previous studies have identified that cosmetic appearance can impact the adoption of assistive technologies in SCI and other health conditions [26, 27]. Regarding device design, “Consumers want assistive devices that reflect their personality” [28]. Additionally, regarding material selection, individuals with SCI are more likely to develop skin issues than those without SCI due to reduced mobility, circulation, sensation, and temperature regulation below the injury [29]. As a result, individuals with SCI are acutely aware of the necessity of preventative skin care and stressed the importance of having device materials that are compatible with their skin [30].

The fourth theme, communication, was prevalent in 85% of the focus groups. Communication was divided into subthemes of PLEX‐oriented language, risks, and outcomes. Communication is important following a SCI, especially during the acute phase when patients and their families are under high levels of psychological distress [31]. Effective communication between patients and healthcare providers is vital for effective patient care and informed consent [32], especially as SCI innovations can be overwhelming and unfamiliar to newly injured individuals [33].

The final two themes were both present in 81% of the focus groups. The first was time, which was split into three sub‐themes: workflow, efficiency, and time commitment. Individuals want to spend less time managing their health and gain back participation in meaningful activities. For example, following a SCI, managing bowel function is a common challenge which can be very time‐consuming and has been reported to have significant restrictions on social activities [34]. In addition, the time to manage a person's health following a SCI also affects a person's ability to work, given they need to manage multiple health conditions and require longer to get ready, often needing assistance [35]. As a result, products that can make people more efficient and self‐manage their health would have a tremendous impact. Conversely, novel products and therapeutics that may improve some aspects of a person's life but increase their time and cost to deploy would be considered an inferior option. For instance, acquiring new devices and therapeutics often requires a prescription which takes additional time and may delay treatment. Streamlining the prescription process could help providers minimize delays.

The second was adaptation considerations, which was divided into three sub‐themes: risk versus benefit, unknown consequences, and current practice. Due to the high risk of secondary complications following SCI, safety is essential when PLEX consider new devices and therapeutics [36, 37]. The adaptation considerations of PLEX was influenced by the time since injury and level of injury. There was a general agreement that in the acute phase, the risk versus benefit analysis would tolerate slightly more risk if it meant possible improvement. Individuals with chronic SCI did not want to risk adverse complications by changing working routines such as bowel care, where there is the risk of losing independence or having a bowel accident [38]. Another fundamental aspect of resistance to change is that once people have learned to adapt to using a given technique or strategy to accomplish a task, they are often unwilling to use a new innovations due to the time and effort it took initially to master the current strategy, especially if the alternative will require significant time and effort to learn or if the result is not guaranteed.

Priorities for the SCI community include interventions and devices that improve quality of life [8]. However, little research has looked at the design of products for the SCI community. There is literature on patient engagement in healthcare such as clinical guidelines [39] and preclinical research [40], but to our knowledge very little research has examined PLEX participatory design with commercializing innovation.

The design of the PLEX focus groups described in this paper is consistent with the IKT model [16], where researchers partner with knowledge users to yield relevant and impactful research results [41]. Product development literature has shown that targeted focus groups for pre‐prototype and post‐design phases result in successful innovation development and transfer [42]. The traditional approach in SCI innovation has not prioritized involving the PLEX community or has involved few individuals for sporadic, short periods. The ongoing partnership of the PLEX community with companies throughout all stages of innovation, is the focus of the Praxis Innovation Program [40].

This paper provides an actionable example of how PLEX were engaged and provided feedback on SCI related medical therapeutics. Clinicians, regulators, and industry partners may benefit from this approach. For clinicians, previous studies have shown that patient engagement improves patient compliance [43]. For regulators, recommendations should go beyond encouraging patient/PLEX engagement to mandating it in the development of SCI‐related devices. The focus group methodology provides valuable input for industry partners on the determination of user needs and demands, prototype development with rapid feedback cycles, and marketing approaches. The methodology and process outlined in this paper may help companies obtain input from users that can inform the design of their innovation. Future work is needed to evaluate the long‐term impact of having PLEX meaningfully engaged throughout the research and design and commercialization process.

In considering these findings, it is also important to consider the limitations. Recruitment was conducted using a SCI contact list and social media, which may have favored members of the SCI community already active in advocacy and influenced the diversity of applicants. There were no restrictions on the number of focus groups attended. Therefore, specific themes may have been overrepresented due to repeat participants. There were more participants with chronic SCI than acute SCI, which may have affected the focus group discussions by putting more weight and focus on longer‐term SCI priorities and interests. It is also possible that the participants in the focus groups may not be fully representative of all individuals living with SCI. Additionally, individuals with higher‐level injuries, ventilator dependence, or living in care facilities may have been underrepresented. Future focus groups will continue monitoring equity and diversity metrics to ensure the SCI population is well represented.

## 5 Conclusion

This study describes the results of using a systematic approach and framework for SCI PLEX focus groups during the research, design and commercialization process. The themes identified: purchase and use decisions, purpose, design, communication, time factors, and adaptation considerations, are important topics to consider for commercializing SCI innovations. Future studies should focus on measuring the impact of patient/PLEX engagement by comparing the success of products that successfully reach consumer market after collaboration with PLEX versus products that are developed independently.

## Author Contributions

**Julia T Ross:** formal analysis (lead), writing – original draft (equal), writing – review and editing (equal). **Vanessa K Noonan:** conceptualization (equal), supervision (supporting), writing – original draft (equal), writing – review and editing (equal). **John Chernesky:** methodology (equal), investigation (equal), writing – review and editing (equal). **James Hektner:** investigation (equal), writing – review and editing (equal). **Richard Peter:** investigation (equal), writing – review and editing (equal). **Spring Hawes:** investigation (equal), writing – review and editing (equal). **Andrew Forshner:** funding acquisition (supporting), methodology (equal), writing – review and editing (equal). **Shweta Shekhar:** data curation (equal), project administration (equal), writing – review and editing (equal). **Tathagata Ray:** data curation (equal), project administration (equal), writing – review and editing (equal). **Arushi Raina:** conceptualization (equal), funding acquisition (lead), supervision (lead), validation (equal), project administration (equal), writing – review and editing (equal). **James J Laskin:** formal analysis (supporting), methodology, validation (equal), supervision (supporting), writing – original draft (equal), writing – review and editing (equal).

## Ethics Statement

The recruitment protocol, focus group methodology, and informed consent used for the SCI Accelerate and Incubate Programs were approved by the Veritas Independent Review Board (Kirkland, Quebec, Canada): 2023‐2480‐16041‐3.

## Consent

All participants signed an electronic consent form to participate in the study and publication disclosures allowing the data collected to be published.

## Conflicts of Interest

The authors declare no conflicts of interest.

## Supporting information

Appendix.

## Data Availability

Queries on the data of this study can be directed to the corresponding author. The data are not publicly available due to privacy or ethical restrictions.
