# Supplementary material for: Engagement of People With Lived Experience in Spinal Cord Injury to Address Innovation Priorities
Source: Health Expect. 2025 Aug 22;28(4):e70369. doi: 10.1111/hex.70369 (PMC12373396; doi:10.1111/hex.70369)
Supplement: Supplementary file 1 — Appendix. [file HEX-28-e70369-s001.docx]

**Appendix:**

A.1 COREQ Checklist

| **Domain 1: Research team and reflexivity** |  |  |
| --- | --- | --- |
| Personal Characteristics |  |  |
| 1. | Interviewer/facilitator | Members of PLEX team facilitated focus groups |
| 2. | Credentials | *PhD, PT, BSc* |
| 3. | Occupation | Members of Praxis |
| 4. | Gender | Both male and female researchers. |
| 5. | Experience and training | Researchers had experience with SCI innovation, research, and clinical practice. |
| Relationship with participants |  |  |
| 6. | Relationship established | Participants were aware that the goal of the focus groups was to address questions for company’s SCI product development. |
| 7. | Participant knowledge of the interviewer | Participants were aware the facilitator was a personal with lived experience. |
| 8. | Interviewer characteristics | Interest in the research topic due to lived experience with SCI |
| **Domain 2: study design** |  |  |
| Theoretical framework |  |  |
| 9. | Methodological orientation and Theory | Conventional content analysis |
| Participant selection |  |  |
| 10. | Sampling | Purposive sampling |
| 11. | Method of approach | Over email from a contact list and on social media. |
| 12. | Sample size | 198 |
| 13. | Non-participation | Two participants left during the focus group discussions. One due to a scheduling conflict and the other due to an unstable internet connection. |
| Setting |  |  |
| 14. | Setting of data collection | Online |
| 15. | Presence of non-participants | Researchers (muted and video off) and facilitators were present |
| 16. | Description of sample | Diversity in injury level, years of SCI, location, sex, and age. |
| Data collection |  |  |
| 17. | Interview guide | Discussion guide created by researchers and the company whose product was being discussed. |
| 18. | Repeat interviews | None |
| 19. | Audio/visual recording | Otter AI, a virtual transcription service used recording to make de-identified transcripts. Once the transcripts were complete the recording was destroyed. |
| 20. | Field notes | Field notes were taken by researchers observing the focus groups |
| 21. | Duration | 90 minutes |
| 22. | Data saturation | Data saturation was determined once themes were present in over 80% of the focus groups. |
| 23. | Transcripts returned | Transcripts were not returned to participants. |
| **Domain 3: analysis and findings** |  |  |
| Data analysis |  |  |
| 24. | Number of data coders | An independent researcher not present in the focus groups coded the themes. |
| 25. | Description of the coding tree | No |
| 26. | Derivation of themes | Themes were derived from the data in the focus group summaries. |
| 27. | Software | Coding was done manually |
| 28. | Participant checking | The facilitators and focus group researchers provided feedback to the independent researcher. |
| Reporting |  |  |
| 29. | Quotations presented | Quotes were presented without any identification. |
| 30. | Data and findings consistent | Yes |
| 31. | Clarity of major themes | Major themes were present in over 80% of the focus groups. |
| 32. | Clarity of minor themes | The minor themes are discussed with quotes and examples. |
